# Oral language comprehension of young adults with low-level reading comprehension

**DOI:** 10.3389/fpsyg.2023.1176244

**Published:** 2023-08-02

**Authors:** Irit Bar-Kochva, Réka Vágvölgyi, Josef Schrader, Hans-Christoph Nuerk

**Affiliations:** ^1^German Institute for Adult Education (DIE) - Leibniz Centre for Lifelong Learning, Bonn, Germany; ^2^Department of Educational and Social Science, Faculty of Human Sciences, University of Cologne, Cologne, Germany; ^3^Center for Cognitive Science, Department of Cognitive and Developmental Psychology, University of Kaiserslautern-Landau, Kaiserslautern, Germany; ^4^Institute of Education, University of Tübingen, Tübingen, Germany; ^5^LEAD Graduate School & Research Network, University of Tübingen, Tübingen, Germany; ^6^Department of Psychology, University of Tübingen, Tübingen, Germany

**Keywords:** reading, low literacy skills, listening comprehension, oral language, adult skills, transparent orthography

## Abstract

Significant difficulties in reading comprehension, despite attendance of compulsory schooling, are a worldwide phenomenon. While previous research on adults with low literacy skills focused primarily on their reading ability, less is known about their oral language skills. In this *Brief Research Report*, we present an investigation of the listening comprehension skills of a selected group of German-speaking young adults, whose reading comprehension is at a primary school level (*n* = 32, ages 16 to 19  years). In addition, the relationship between listening comprehension and reading comprehension, beyond word reading skills, was tested. Standardized tests of reading and listening comprehension in the German language were administered. The average performance of the group in the listening comprehension tasks was below the level expected by age and educational level. In addition, when entered into a stepwise regression equation, listening comprehension, but not word reading, explained a significant amount of variance in reading comprehension. This pattern of relationship differs from previous findings in studies of adults struggling to read the opaque English orthography. Whether orthographic transparency explains this discrepancy should be further tested in cross-orthography studies with larger samples of adults with low literacy skills.

## Introduction

Large-scale surveys indicate high rates of adults with low literacy skills in the OECD countries. According to the PIAAC survey, an average of 18.9% of the adults across the participating countries have low reading skills (Level 1 or below according to the surveys reading literacy scale, see OECD, 2016). The PISA survey of 15-year-old students indicates a similar problem (Schleicher, 2019). The ability to comprehend written texts at a reasonable pace has a central role in defining the functionality of reading (UNESCO, 1978; Egloff et al., 2011; Vágvölgyi et al., 2016). Accordingly, reading level is typically assessed in these surveys using tasks that require comprehension of written texts.

Reading comprehension and listening comprehension share many aspects (Clifton et al., 2003). Both are highly complex procedures, which involve a line of sub-processes, that must be carried out effectively and with good synchronization. For instance, both involve sub-lexical, lexical and syntactic processing, as well as processes of the working memory (Perfetti et al., 2005; Imhof, 2010; Kim and Pilcher, 2016). In addition, both require the creation of a mental representations of the text, which is constantly being updated with new inputs (Hemforth and Konieczny, 2006). Yet the modes of text communication also impose distinct demands of processing, such as the need to decipher the script in reading, the possibility to relate to vocal cuing while listening, and the ease with which texts can be returned to in reading compared to oral communication. The question arises whether the comprehension difficulties of adults with low literacy skills, for which research usually focuses on reading comprehension, in fact involve both the written and the oral modes of text communication.

The close relations between reading comprehension and listening comprehension have already been modeled by Gough and Tunmer’s (1986) Simple View of Reading (SVR) in a concise manner. The model predicts that the variance in reading comprehension could be explained to a large degree with only two measures: (1) accuracy or fluency in decoding (examined with word or pseudoword reading) and by (2) listening comprehension (Hoover and Gough, 1990). Regarding the first component (decoding), adults with low literacy skills seem to have, on average, significant difficulties reading words and pseudowords (Greenberg et al., 1997; Thompkins and Binder, 2003; Winn et al., 2006; Mellard et al., 2010; Nanda et al., 2010; Grosche and Grünke, 2011; Mellard and Fall, 2012; Boltzmann and Rüsseler, 2013; Bar-Kochva et al., 2019). As concerns the second component (listening comprehension), deficits in some sub-processes of language comprehension, which may influence both reading and listening comprehension, have been reported for these adults, including phonological working memory, vocabulary and syntactic processing (Greenberg et al., 1997; Sabatini et al., 2010; Grosche and Grünke, 2011; Taylor et al., 2012). The level of listening comprehension among adults with low literacy skills has, however, been rarely examined, and the available studies vary considerably in their results. Some reported very low level of listening comprehension, equivalent to 4th and 5th grade levels (Sabatini et al., 2010; Tighe et al., 2023). Other studies suggest a somewhat better average performance (7th-10th grade level), but still below the level expected by age (Braze et al., 2007, 2016; Barnes et al., 2017). A higher average performance in a listening comprehension task, which lies at the 37th percentile according to norms, has been reported by Mellard et al. (2010). One possible explanation for this broad variance of the results is that studies of adults with low literacy skills often include participants attending various types and levels of basic and language education courses. This recruitment procedure may naturally lead to a considerable heterogeneity in various aspects. An examination of more homogenous subgroups may be needed in order to better understand the listening comprehension skills of low literate adults.

Studies of adults with low literacy skills confirm the relations between both decoding and listening comprehension with reading comprehension (Mellard et al., 2010; Sabatini et al., 2010; Mellard and Fall, 2012; Tighe and Schatschneider, 2016). The study by Mellard and Fall (2012) suggests, however, that decoding is a strong predictor of reading comprehension across reading levels among adults in the need of basic or secondary education, whereas the contribution of listening comprehension becomes significant once a certain threshold of reading skills has been reached (also see the study by Talwar et al., 2021). These results are in line with developmental studies of the SVR (Catts et al., 2005). However, in these studies, readers of English orthography were examined, which is an orthography with inconsistent grapheme-phoneme relations. This characteristic of English has been found to constitute a source of difficulty in processes of decoding/word reading (Share, 2008). In contrast, transparent orthographies, with more consistent relations between spelling and sound, have been found to facilitate the fast acquisition of decoding/word reading skills, even in children with a reading disability (Wimmer, 1993; Landerl et al., 1997). This suggests that the required threshold of decoding/word reading ability is more easily met when reading transparent orthographies, which may leave more variance in reading comprehension to be explained by listening comprehension.

It should be noted that a large body of research of children who read different languages and orthographies has contributed significantly to the understanding of the universal and the orthography-dependent aspects of reading acquisition and reading disability (Share, 2008). However, research on adults with low literacy skills, who read orthographies other than English is scarce. In addition, current models of reading, which are mainly based on the study of children, may not be fully generalized to adults with low literacy skills (Mellard et al., 2010). Further research of adults with low literacy skills, who struggle with reading orthographies other than English, should contribute to the understanding of the phenomenon of low literacy skills in different linguistic environments.

### The current study

This *Brief Research Report* adds to previous studies by relating to a group of young adults struggling with reading comprehension of German, which has a transparent orthography in reading. In addition, an effort has been made to create a more homogeneous sample compared to previously examined samples in terms of age range, educational background, current educational context, estimated general ability, and diagnosed neurological conditions. The following questions and hypotheses were formulated:

1. Do young adults with low reading comprehension who read a transparent orthography like German show a deficit in listening comprehension?

Considering that reading comprehension and listening comprehension share many cognitive processes (irrespective of the orthography being read), young adults with low reading comprehension skills who read German were expected to present deficits in listening comprehension, as has been previously reported in readers of English.

1. To which extent does listening comprehension explain the variance in reading comprehension beyond decoding/word reading in these young adults?

Due to the transparent nature of German orthography in reading, we expected to find a significant contribution of listening comprehension to explaining the variance in reading comprehension beyond the contribution of decoding/word reading.

## Method

### Participants

Thirty-two participants (20 men) were selected from a database previously collected by the second author of this *Brief Research Report* (Vágvölgyi, 2018). The original database included 191 adults attending basic education courses or vocational trainings. Other studies relating to this database, which address different questions from the ones explored in the present study, appear in Vágvölgyi et al. (2019) and Bar-Kochva et al. (2021). The entire sample proved to be heterogeneous in many aspects, including in terms of reading comprehension level. Hence, a first selection criterion was a reading comprehension score which was below the level expected from 6th graders (ELFE 1–6, Lenhard and Schneider, 2006). Notably, reading comprehension, which is at or below the level expected from primary school children, has been suggested as an operational definition of low literacy skills in adulthood (Grosche and Grünke, 2011). Since many countries have six years of primary school, the 6th grade was chosen as the cut-off point. In addition, with the aim of reducing effects of distance from formal education as well as of other possible age-related factors, only young participants attending vocational training were included in the current analysis (age range 16–19 years, mean age = 16.75 years, SD = 0.762). These participants spent between nine and twelve years in school, with a mean of 10.94 years (SD = 0.84). One half of the sample repeated a class once or twice. To reduce effects of lack of opportunity to attend the German educational system from an early age, only participants born in Germany were included. When asked about the language spoken at home, six participants reported speaking a language other than German, 12 reported speaking German and another language, and 14 spoke only German. Due to the overrepresentation of people who speak German as a second language among adults with low literacy skills (Grotlüschen and Buddeberg, 2020), we did not exclude participants who did not speak German as their first language, preferring to refer to a screening criterion related to the opportunity to attend the German-speaking educational system from an early age. To control for specific cases of low performance in literacy tasks, which may be explained by low IQ or a special diagnosed neurological condition, participants were included provided they had an IQ approximation score (based on a non-verbal processing speed test from the LPS-2 battery, Kreuzpointner et al., 2013) within the average range and did not report of a known diagnosed hearing deficit, uncorrected sight problem or attention deficit. A diagnosed language disability or a learning disability/difficulty was not an exclusion criterion, as these conditions are expected in adults with low literacy skills (Eme, 2011). At the same time, none of the 32 participants reported such diagnoses. Notably, an approximation of only 13% of the original sample (N = 191) reported at least one diagnosis, and only 4.2% reported a diagnosis in the field of reading (dyslexia or a reading difficulty or disability). As these proportions are not in accordance with the low literacy level of the participants (Gottesman et al., 1996), it is assumed that the population examined is an underdiagnosed one. Hence the self-reported background information should be treated with caution. The mean IQ approximation score of the group was 93.52 (SD = 8.71).

### Materials

In this *Brief Research Report*, we relate to the tasks, which were relevant to the examination of the research questions. For the complete set of tasks included in the original database, see Vágvölgyi (2018). The selected tests are commonly used in Germany in both research and diagnostic work. These were evaluated for different types of validity, that proved to be satisfactory (detailed validity analyses appear in the tests’ manuals). Reliability coefficients are mentioned next to each test.

#### IQ approximation score

Test number three from the standardized *Leistungsprüfsystem 2* battery (LPS-2, Performance Testing System, Kreuzpointner et al., 2013) was used. In this subtest, one odd symbol must be located within a row of symbols within a given duration. The battery was designed to examine different dimensions of intelligence (crystalized intelligence, fluid intelligence, visual perception and cognitive speed, as well as g), and the test manual allows for a conversion of subtest scores into an estimated full IQ score. Nevertheless, as a complete IQ evaluation was not possible in this study, the estimated full IQ score is considered here with caution and served only for the purpose of screening participants with an IQ approximation score which is below 80. Reliability coefficients are between *r* = 0.77 and *r* = 0.89.

#### Reading comprehension

The comprehension of short texts was examined using the standardized *Leseverständnistest für Erst- bis Sechstklässler* (ELFE 1–6, Reading Comprehension Test for 1st to 6th graders, Lenhard and Schneider, 2006). The test was designed to capture a broad range of reading comprehension skills of children between the 1st and the 6th grades. Accordingly, it was estimated that it would capture the variance within a group of low literate adults, and concurrently avoid a floor or a ceiling effect. The text reading subtest included 20 short texts, each followed by multiple-choice comprehension questions. Working time was limited to six minutes. The reliability coefficient is 0.92.

#### Decoding

Pseudoword- and word reading were tested using two subtests from the standardized SLRT-II (*Salzburger Lese-und Rechtschreibtest II*, Salzburg reading and spelling test II, Moll and Landerl, 2010). Each subtest included a list of 156 pseudowords or words, which were presented in an ascending order of difficulty. Participants were asked to read out aloud as many items as they could within one minute. Reliability coefficients are between 0.90 and 0.98.

#### Listening comprehension

Two subtests from the standardized ADST (*Allgemeiner Deutscher Sprachtest*, General German language test, Steinert, 2011) were administered. These loaded on the same factor, and showed high correlations with results from the entire test battery (above *r* = 0.80).

##### Oral story comprehension

The subtest addresses the ability to understand language at the level of texts. Participants heard two recorded short stories followed each by five comprehension yes/no questions relating to the contents of the texts. Each story was played only once. The total recording duration was 5:28 min. Both stories described a conversation between two people. The questions addressed information, which is explicitly mentioned in the texts, as well as global understanding of the texts. A short text with three questions preceded the two test stories as an example. The reliability coefficient of this subtest is 0.80.

##### Oral grammatical comprehension

The subtest addresses the language level of sentence grammatic. Participants listened to a recording of single complex sentences, each followed by a comprehension question. The sentences were played once, and the total recording duration was 3:41 min. Two events were presented in each sentence. Participants were asked to judge whether an event occurred before, after, or at the same time as another event mentioned in the same sentence, e.g., *When the rain stopped, they went for a walk. When did the rain stop?* Possible answers: *Before, after or at the same time* (as the second event). Three examples preceded the task, and the test items included 10 sentences. Reliability coefficient of this subtest is 0.73.

### Procedure

The participants gave their written informed consent to take part in a study on the language skills of young adults in vocational trainings and in adult education. One-hundred and fifty Euros were paid to the participants’ class-funds as a compensation for the time invested in the study. The students were tested in their education institution, and the order of task administration was the same for all participants. A short oral interview, in which background information on participants was collected, preceded the administration of the standardized tests. All tasks presented in this *Brief Research Report*, except for the listening comprehension tasks, included parallel forms. Students were randomly assigned to one of the forms of these tests. The administration of all the tests in the original study was divided between two sessions, each lasting approximately one and a half hours. The completion of the tasks included in the present analysis required approximately 25 minutes.

## Results

In Table 1, the tests’ raw scores are presented, as well as their conversion to percentile ranges or standardized scores based on available norms. The test of pseudoword- and word reading included norms of young adults (with students in vocational trainings represented), and hence achievements in these tests could be compared to the expected performance in the broad population of young adults. However, the listening comprehension tests included norms only of school-children and of adolescents attending the different types of schools in the German educational system. Performance was therefore compared to the norms of students of the two lower-level schools in Germany, the one leading to a graduation of either 9 years of schooling with a possibility to continue to a tenth year (*Hauptschule*), and one that leads to a graduation of 10 years of schooling (*Realschule*). Upon completion of these two types of schools, students often continue to vocational trainings. Hence this choice of reference-norms seemed a reasonable compromise. It may also be noted, that in order to have a common reference of comparison to norms of students from *Hauptschulen* and *Realschulen*, norms of 9th graders were used for comparison, as attendance in this class is still mandatory in both types of schools.

**Table 1:** Performance in the components of the SVR model (reading comprehension, decoding/word reading and oral comprehension) presented in raw scores and in percentile ranges or standard scores.

Test	Measure	Minimum	Maximum	Mean	SD
ELFE 1–6 Reading comprehension	Raw score in text reading (number of questions answered correctly out of 20)	0.00	14.00	11.37	2.89
SLRT II Word reading fluency	Raw score (words correctly read within 1 min).	42.00	119.00	84.69	20.63
	Percentile range based on adults’ norms	<1	42–62	3–7	–
	% Errors	0.00	14.29	2.77	3.76
SLRT II Pseudoword reading fluency	Raw score (pseudowords correctly read within 1 min).	22.00	85.00	53.19	15.47
	Percentile range based on adults’ norms	<1	71–75	12–16	–
	% Errors	0.00	33.33	5.62	7.04
ADST Oral story comprehension	Raw score (correct answers out of 10)	3.00	9.00	6.34	1.73
	Standard score based on norms of 9th graders in *Hauptschulen*	−4.58	0.42	−1.80	1.44
	Standard score based on norms of 9th graders in *Realschulen*	−4.75	0.25	−1.96	1.44
ADST Oral grammatical comprehension	Raw score (correct answers out of 10)	1.00	10.00	5.53	2.61
	Standard score based on norms of 9th graders in *Hauptschulen*	−4.19	1.44	−1.35	1.63
	Standard score based on norms of 9th graders in *Realschulen*	−5.85	1.08	−2.36	2.01

Participants performed on average very poorly in the reading tasks, reaching percentiles 3rd-7th in word reading fluency and 12th-16th in pseudoword reading fluency (see Bar-Kochva et al., 2021 for a more detailed analysis of difficulties of low literate adults in these measures). The average accuracy level was very high in these two tests, as would be expected from readers of a transparent orthography. As reading fluency was suggested to be a more sensitive measure than reading accuracy among readers of transparent orthographies (Wimmer, 2006), fluency in pseudoword and word reading was used for further analysis. The average level of performance in the two oral comprehension tasks was below the level expected by 9th graders in the two school types. When compared to norms of students in *Hauptschulen*, participants reached a mean score which is 1.80 and 1.35 standard deviations below the mean in story comprehension and in grammatical comprehension, respectively. When compared to norms of students in *Realschulen*, participants reached a mean score which is 1.96 and 2.36 standard deviations below the mean in story comprehension and in grammatical comprehension, respectively. In view of the previously reported broad variance characterizing adults with low literacy skills (e.g., Mellard and Fall, 2012), in an additional analysis, it was examined whether low-level listening comprehension is shared by all participants. Supplementary Table S1 in the Supplementary Material indicates that the majority of participants had a score which is below the mean score expected from 9th graders in the two forms of schools.

Pearson correlation coefficients between reading comprehension and its components according to the SVR model were further calculated (Table 2). The only measures significantly correlating with reading comprehension were oral grammatical comprehension and word reading. Based on these results, a linear stepwise regression analysis was designed, with reading comprehension as a dependent variable, and word reading and oral grammatical comprehension as independent variables. In order to explore to which extent listening comprehension explains the variance in reading comprehension beyond word reading skills, word reading was entered in a first step as an independent variable, and oral grammatical comprehension was entered in a second step (Table 3). Table 2 indicates that there was no multicollinearity between the independent variables. An examination verifying that the regression assumption of homoscedasticity was met, appears in Supplementary Figures S1–S3. The analyses indicate that the distribution of residual scores resemble a normal distribution. The regression model was significant *F* change (2,29) = 6.101, *p* = 0.006. Word reading explained 14% of the variance (*p* = 0.035) when entered in step 1, and the inclusion of oral grammatical comprehension in step 2 explained an additional 15.6% of the variance (*p* = 0.017). The contribution of word reading to explaining the variance in reading comprehension in step 2 was no longer significant (*β* = 0.272, *p* = 0.101).

**Table 2:** Pearson correlation coefficients between the components of the SVR model (reading comprehension, decoding/word reading and oral comprehension).

	Reading comprehension correct items	Pseudoword reading fluency	Word reading fluency	Oral story comprehension	Oral grammatical comprehension
Reading comprehension correct items	1				
Pseudoword reading fluency	0.268	1			
Word reading fluency	0.374^*^	0.779^**^	1		
Oral story comprehension	0.134	−0.136	0.025	1	
Oral grammatical comprehension	0.476^**^	0.222	0.248	0.257	1

^*^Correlation is significant at the 0.05 level (2-tailed). ^**^Correlation is significant at the 0.01 level (2-tailed).

**Table 3:** Coefficients of a linear stepwise regression analysis predicting the variance in reading comprehension with word reading and oral grammatical comprehension.

		*B*	SE	β	*t*	*p*
Step 1	Word reading fluency	0.052	0.024	0.374	2.207	0.035
Step 2	Word reading fluency	0.038	0.023	0.272	1.694	0.101
	Oral grammatical comprehension	0.452	0.178	0.408	2.539	0.017

## Discussion

Two research questions were explored. First, we examined whether young adults with low reading comprehension who read a transparent orthography have low-level listening comprehension as well. On average, participants performed below the level expected from 9th graders attending schools which lead to the completion of either 9 or 10 years of schooling. The fact that age-appropriate norms were unavailable restricted the interpretation of the results. Nevertheless, it may at the minimum be argued, that in line with the study’s prediction, the average listening comprehension skills of the group examined were below the level expected by age and educational level. These results are in line with previous studies of low literate adults who read English (e.g., Sabatini et al., 2010).

At the same time, not all participants in the sample showed low performance in the listening comprehension tasks. The heterogeneity of adults with low literacy skills in terms of social-background, cognitive as well as in reading skills has been repeatedly reported (e.g., Gottesman et al., 1996). As mentioned in the Introduction, the recruitment procedure of adults with low literacy skills in previous studies may have led to a broad variance in various measures. Moreover, it is often difficult to assess whether adequate learning opportunities were provided in childhood when examining adults. However, this information is essential in the diagnosis of a learning disability (Lyon et al., 2003). As a result, samples of adults with low literacy skills may include varying levels of difficulties which may also have different sources. In the present investigation, we examined a more homogenous group of adults with low literacy skills, yet a notable variance in listening comprehension skills was still evident.

The second question examined in this study addressed the contribution of listening comprehension to explaining the variance in reading comprehension beyond the contribution of decoding/word reading. In line with the study’s prediction, listening comprehension contributed significantly to explaining the variance in reading comprehension. The finding that the contribution of word reading was insignificant once listening comprehension entered the equation is in contrast to previous reports from studies of English readers with low literacy skills (Mellard and Fall, 2012; Talwar et al., 2021). The accuracy rate in word reading was very high, as would be expected from readers of a transparent orthography, even in the presence of a reading disability (Wimmer, 1993). As fluency in word reading includes the factor of accuracy in addition to speed, this measure may have had less variance than the same measure for English readers. Consequently, its role in explaining the variance in reading comprehension may have also been reduced. At the same time, whether orthographic transparency explains these discrepant results should be verified in a cross-orthography study with larger samples.

The finding that only one of the two listening comprehension tasks correlated with reading comprehension requires further thought. The story comprehension task, which did not correlate with reading comprehension, addressed the understanding at the text level, including the retrieval of information explicitly indicated in short texts, as well as global understanding of these texts. The oral grammatical comprehension task, which did correlate with reading comprehension, addressed the understanding at the syntactic level. The current results may then emphasize the relevance of listening comprehension at the syntactic level to reading comprehension in the studied group. The current data accord with previous findings on deficits at the syntactic level in low literate adults (Taylor et al., 2012).

The implications of this study are considered. The results add to the understanding of the characteristics of adults with low literacy skills by suggesting that their difficulties are not restricted to tasks involving the written language but extend to oral comprehension as well. From a practical perspective, the results point to listening comprehension as a skill that should receive attention in diagnostic procedures and interventions when designing programs for young adults with low literacy skills. Studies of children with learning disabilities as well as of typically developing children indicate that improvements in the components of listening comprehension are possible through explicit and systematic instruction of, for example, oral vocabulary, grammatical knowledge and comprehension strategies (Kim and Pilcher, 2016).

The study has several limitations: The small sample size, the lack of age-appropriate norms for all tests and the limited range of oral language skills addressed in this study. In addition, factors such as SES or developmental disabilities, which were not collected or reported by the participants in the interview, may interact with the results. As the measures available for the current analyses were not originally collected to address the two research questions posed, this study could only provide a first indication of results. These should be further verified in studies specifically designed to address the two research questions using cross-orthography comparisons, larger samples and a wider range of language and background measures.

## Data availability statement

Datasets could be made available on request. Requests to access these datasets should be directed to Hans-Christoph Nuerk, hc.nuerk@uni-tuebingen.de.

## Ethics statement

The studies involving human participants were reviewed and approved by the Faculty of Economics and Social Sciences Ethics Committee of the University of Tübingen. Written informed consent from the participants’ legal guardian/next of kin was not required to participate in this study in accordance with the national legislation and the institutional requirements.

## Author contributions

IB-K conceptualized the secondary data analysis, analyzed the data and wrote the first draft of the manuscript. RV, JS, and H-CN contributed to most aspects involved in the original study for which the complete set of data were collected. All authors contributed to the revision of the first draft of this manuscript, including to its theoretical conceptualization.

## Funding

The research for which the dataset was collected was funded by the LEAD Graduate School & Research Network [GSC1028], a project of the Excellence Initiative of the German federal and state governments and by the German Institute for Adult Education—Leibniz Centre for Lifelong Learning e.V.

## Conflict of interest

The authors declare that the research was conducted in the absence of any commercial or financial relationships that could be construed as a potential conflict of interest.

## Publisher’s note

All claims expressed in this article are solely those of the authors and do not necessarily represent those of their affiliated organizations, or those of the publisher, the editors and the reviewers. Any product that may be evaluated in this article, or claim that may be made by its manufacturer, is not guaranteed or endorsed by the publisher.

## Supplementary material

The Supplementary material for this article can be found online at: https://www.frontiersin.org/articles/10.3389/fpsyg.2023.1176244/full#supplementary-material
