# Supplementary material for: Oral language comprehension of young adults with low-level reading comprehension
Source: Front Psychol. 2023 Aug 2;14:1176244. doi: 10.3389/fpsyg.2023.1176244 (PMC10433201; doi:10.3389/fpsyg.2023.1176244)

Supplementary Material

Table S1. Frequency analysis of performance level in the listening comprehension tasks

Standard score	Frequency	Percent	Cumulative Percent
Oral story comprehension SS *Hauptschule*			
-4.58	2	6.3	6.3
-3.75	4	12.5	18.8
-2.92	3	9.4	28.1
-2.08	7	21.9	50.0
-1.25	8	25.0	75.0
-.42	4	12.5	87.5
.42	4	12.5	100.0
Total	32	100.0	
Oral story comprehension SS *Realschule*			
-4.75	2	6.3	6.3
-3.92	4	12.5	18.8
-3.08	3	9.4	28.1
-2.25	7	21.9	50.0
-1.42	8	25.0	75.0
-.58	4	12.5	87.5
.25	4	12.5	100.0
Total	32	100.0	
Oral grammatical comprehension SS *Hauptschule*			
-4.19	2	6.3	6.3
-3.56	3	9.4	15.6
-2.94	3	9.4	25.0
-2.31	4	12.5	37.5
-1.69	5	15.6	53.1
-1.06	1	3.1	56.3
-.44	5	15.6	71.9

.19	4	12.5	84.4
.81	4	12.5	96.9
1.44	1	3.1	100.0
Total	32	100.0	
Oral grammatical comprehension SS *Realschule*			
-5.85	2	6.3	6.3
-5.08	3	9.4	15.6
-4.31	3	9.4	25.0
-3.54	4	12.5	37.5
-2.77	5	15.6	53.1
-2.00	1	3.1	56.3
-1.23	5	15.6	71.9
-.46	4	12.5	84.4
.31	4	12.5	96.9
1.08	1	3.1	100.0
Total	32	100.0	

Note. The standard scores (SS) were calculated according to norms of 9th graders attending the two types of schools: *Hauptschulen* or *Realschulen*.

Figure 1. Scatter graph of the residual values and the predicted values

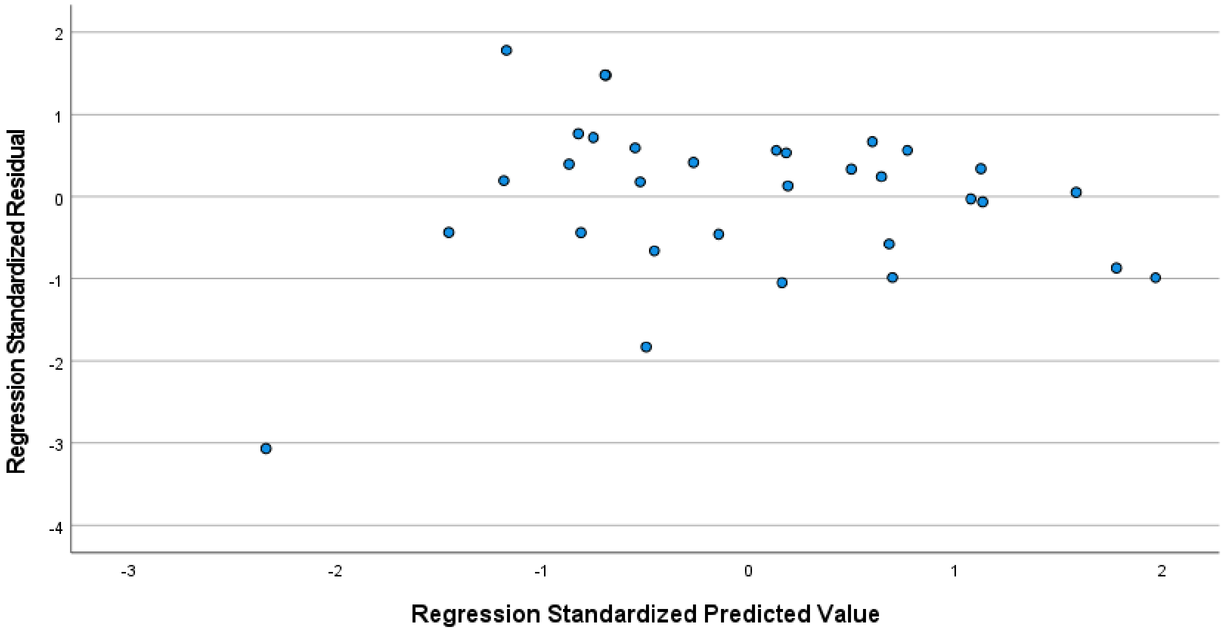

Figure 2. Histogram of distribution of residual scores

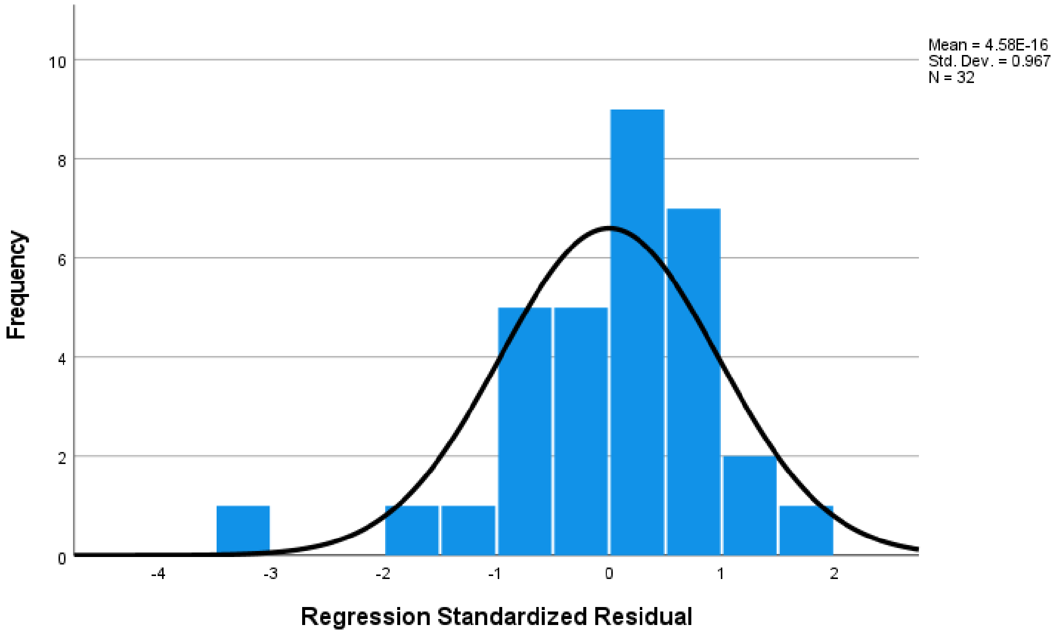

Figure 3. Normal Probability Plot (P-P) of regression standardized residual scores

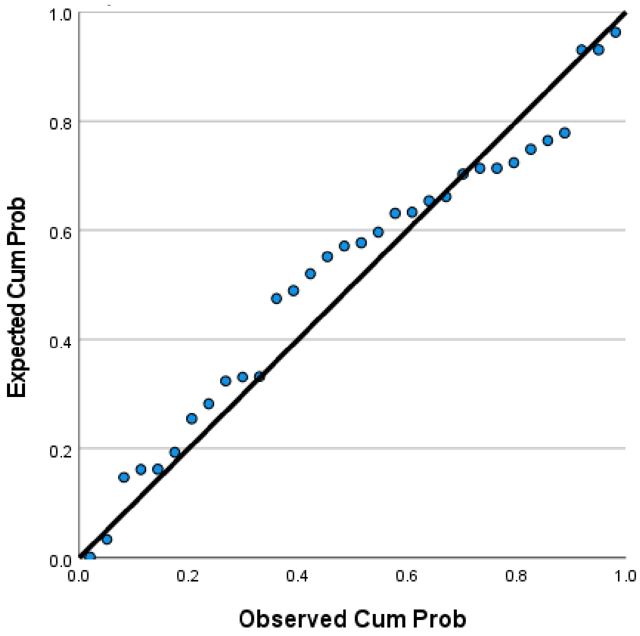

Supplement: Supplementary file 1 [file Data_Sheet_1.pdf]
